# A dominant negative *ADIPOQ* mutation in a diabetic family with renal disease, hypoadiponectinemia, and hyperceramidemia

**DOI:** 10.1038/s41525-022-00314-z

**Published:** 2022-07-22

**Authors:** Christopher A. Simeone, Joseph L. Wilkerson, Annelise M. Poss, James A. Banks, Joseph V. Varre, Jose Lazaro Guevara, Edgar Javier Hernandez, Bushra Gorsi, Donald L. Atkinson, Tursun Turapov, Scott G. Frodsham, Julio C. Fierro Morales, Kristina O’Neil, Barry Moore, Mark Yandell, Scott A. Summers, Andrzej S. Krolewski, William L. Holland, Marcus G. Pezzolesi

**Affiliations:** 1 Department of Human Genetics, University of Utah School of Medicine, Salt Lake City, UT 84112 USA; 2 Division of Nephrology and Hypertension, Department of Internal Medicine, University of Utah School of Medicine, Salt Lake City, UT 84132 USA; 3 Department of Nutrition and Integrative Physiology, University of Utah College of Health, Salt Lake City, UT 84112 USA; 4 Utah Center for Genetic Discovery, Department of Human Genetics, University of Utah School of Medicine, Salt Lake City, UT 84112 USA; 5 Section on Genetics and Epidemiology, Research Division, Joslin Diabetes Center, Boston, MA 02115 USA; 6 Department of Medicine, Harvard Medical School, Boston, MA 02115 USA; 7 Diabetes and Metabolism Research Center, University of Utah School of Medicine, Salt Lake City, UT 84108 USA

**Keywords:** Genetic linkage study, Mutation

## Abstract

Adiponectin, encoded by *ADIPOQ*, is an insulin-sensitizing, anti-inflammatory, and renoprotective adipokine that activates receptors with intrinsic ceramidase activity. We identified a family harboring a 10-nucleotide deletion mutation in *ADIPOQ* that cosegregates with diabetes and end-stage renal disease. This mutation introduces a frameshift in exon 3, resulting in a premature termination codon that disrupts translation of adiponectin’s globular domain. Subjects with the mutation had dramatically reduced circulating adiponectin and increased long-chain ceramides levels. Functional studies suggest that the mutated protein acts as a dominant negative through its interaction with non-mutated adiponectin, decreasing circulating adiponectin levels, and correlating with metabolic disease.

## Introduction

Adiponectin is an adipocyte-derived hormone with pleiotropic actions that promote insulin sensitivity, inhibit cell death, and decrease inflammation^1^. Adiponectin forms an obligate trimer and circulates as trimers, hexamers, and high-molecular weight multimers that target various tissues and cell types, including liver, kidney, cardiac myocytes, and pancreatic β cells. Levels of adiponectin are decreased in obesity and may contribute to a chronic state of inflammation that leads to insulin resistance, type 2 diabetes, coronary artery disease, myocardial infarction, nonalcoholic steatohepatitis, and kidney disease^2–4^. Recently, the antiapoptotic, insulin-sensitizing, glucose lowering, anti-inflammatory, and anti-steatotic effects of adiponectin have been linked to its role in sphingolipid metabolism and its receptor-mediated activation of ceramidase activity, which reduce levels of lipotoxic ceramides^5,6^.

Previous studies have identified common variants in or near *ADIPOQ*, the gene that encodes adiponectin, that are associated with aberrant adiponectin levels^7^, obesity^8^, type 2 diabetes^7,9^, and diabetic kidney disease^10,11^. Rare *ADIPOQ* amino acid substitution mutations have also been reported in individuals with diabetes and hypoadiponectinemia^12,13^. Here, we describe the first multigenerational family with a protein-truncating mutation in *ADIPOQ* (p.Gly93GlufsTer73), diabetes, and end-stage renal disease. Carriers of this mutation have dramatically reduced circulating adiponectin and increased long-chain ceramide levels. Functional studies show that this mutation acts via a dominant negative mechanism, with the wild-type and mutant proteins interacting, leading to decreased levels of cellular and secreted adiponectin. Our findings support adiponectin’s protective role and suggest that its genetic loss leads to ceramide accumulation which, in turn, contributes to diabetes and progression of renal disease.

## Results

### Identification of ADIPOQ as a candidate gene for diabetes and end-stage renal disease

**Fig. 1:**
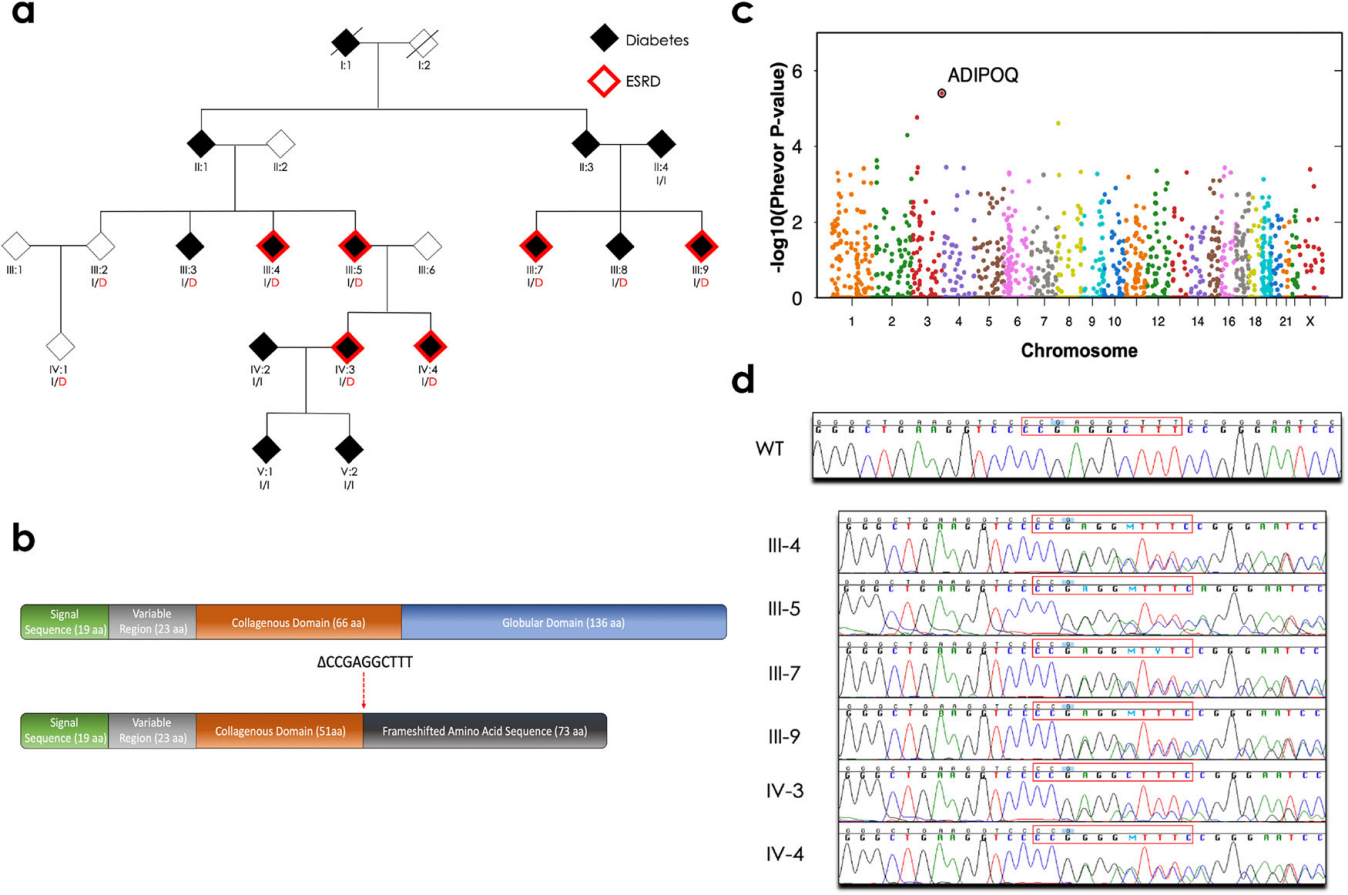
Pedigree of the family enriched for diabetes and end-stage renal disease and identification of the ADIPOQ mutation. **a** The family pedigree, status of diabetes (shaded) and end-stage renal disease (ESRD; red outline), and the *ADIPOQ* mutation (p.Gly93GlufsTer73). Carriers of the wild-type *ADIPOQ* insertion (I) or mutated *ADIPOQ* deletion (D; red) are indicated. **b** The structure of the wild-type (top) and mutant (bottom) adiponectin protein. Wild-type adiponectin monomers consist of a 244 amino acid protein composed of four domains; an N-terminal signal sequence (19 amino acids), a variable region (23 amino acids), a collagenous domain (66 amino acids), and a C-terminal globular domain (136 amino acids). The 10-nucleotide deletion (CCCGAGGCTTT→C, indicated as ∆CCGAGGCTTT) at amino acid 93 creates a frameshift that truncates the adiponectin protein and generates a novel peptide that terminates 73 amino acids after this deletion. **c** The PHEVOR plot using Human Phenotype Ontology (HPO) terms and connections to Gene Ontology terms to prioritize potentially damaging alleles, using terms for a) kidney disease: HP:0000077 (Abnormality of the kidney), HP:0000112 (Nephropathy), and HP:0003774 (Stage 5 chronic kidney disease), and b) diabetes: HP:0000819 (Diabetes) and HP:0005978 (Type 2 diabetes mellitus) and a combination of the pVAAST *p* values and PHEVOR scores. PHEVOR scores (*y*-axis) for each gene (dot) are plotted across the genome (*x*-axis, chromosomes 1-Y). **d** The chromatogram from Sanger sequencing of a non-carrier from the family (WT) and the 6 carriers identified through whole-genome sequencing and pVAAST/PHEVOR analysis.

The goal of this study was to identify a potential genetic cause of disease in a mutigenerational family with diabetes and prominent end-stage renal disease. Included among the cases in this family were three affected sibling pairs, two affected parent-offspring pairs, two affected first-cousin pairs, and two pairs of affected first-cousins-once-removed (Fig. 1a and Table 1). Using unified linkage analysis and rare variant association testing, where the enrichment of rare variants among these cases was compared to 524 ethnically matched background controls, we identified a heterozygous 10-nucleotide deletion in exon 3 of *ADIPOQ* (chromosome 3: 186,572,030; CCCGAGGCTTT→C) shared by all six affected family members (Fig. 1a–d, Supplementary Figs. 1–3, and Supplementary Tables 1–4). This rare deletion (p.Gly93GlufsTer73) was seen only once among 56,810 exome and genome sequences from non-Finnish Europeans reported in the Genome Aggregation Database^14^ and creates a frameshift that results in premature truncation of the adiponectin protein, generating a novel peptide that includes 73 abnormally coded terminal amino acids (Fig. 1b and Supplementary Fig. 4).

**Table 1:** Clinical characteristics and mutation status of family members.

Family Member	Age at Examination	Diabetes	BMI	Age at Diabetes Diagnosis	Duration of Diabetes	Diabetes Treatment	Albumin: Creatinine Ratio (μg/mg)	ESRD	Hypo-adiponectinemia	Hyper-ceramidemia	HLA Status^c^	*ADIPOQ* Mutation Status
II:4	>60	Yes	40.1	<40	51	Oral	34.2	No	No	Yes	X/X	WT
III:2	>60	No	24.6	–	–	–	14.8	No	Yes	Yes	X/X	MUT
III:3	>60	Yes	27.3	>60^a^	–	Unknown	6.7	No	Yes	Yes	X/X	MUT
III:4	>60	Yes	25.6	<40	32	Oral	>2500	Yes	Yes	Yes	X/X	MUT
III:5	>60	Yes	29.2	<40	44	Insulin	313.3	Yes^b^	Yes	Yes	X/X	MUT
III:7	>60	Yes	31.2	40–60	13	Oral	>2500	Yes	Yes	No	X/X	MUT
III:8	40–60	Yes	31.4	40–60^a^	0	Diet	28.3	No	Yes	Yes	DR4/X	MUT
III:9	>60	Yes	33.2	<40	35	Insulin	22.4	Yes^b^	Yes	Yes	DR4/X	MUT
IV:1	40–60	No	35.5	–	–	–	2.6	No	Yes	Yes	X/X	MUT
IV:2	<40	Yes	36.7	<40^a^	0	Diet	39.3	No	No	No	DR3/4	WT
IV:3	40–60	Yes	34.5	<40	25	Insulin	219.9	Yes^b^	Yes	Yes	X/X	MUT
IV:4	<40	Yes	31.3	<40	16	Insulin	325.6	Yes^b^	Yes	Yes	DR3/X	MUT
V:1	<40	Yes	37.8	<40^a^	0	Oral	16.2	No	No	No	DR4/X	WT
V:2	<40	Yes	26.9	<40^a^	1	Insulin	5.9	No	No	No	DR4/X	WT

Data are from baseline clinical characteristics collected at time of enrollment to the Joslin Study on the Genetics of Type 2 Diabetes except as noted. *ADIPOQ* mutation non-carriers (WT); *ADIPOQ* mutation carriers (MUT).

^a^These family members had a diagnosis of diabetes either shortly before enrollment to the Joslin Study on the Genetics of Type 2 Diabetes (III:8) or following their participation in this study (III:3, IV:2, V:1, V:2).

^b^These family members had either normoalbuminuria (III:9) or proteinuria (III:5, IV:3, and IV:4) at enrollment to the Joslin Study on the Genetics of Type 2 Diabetes and later progressed to end-stage renal disease (ESRD).

^c^Human leukocyte antigen (HLA) alleles DR3 (haplotype DQA1*05:01-DQB1*02:01) and DR4 (haplotype DQA1*03-DQB1*03:02) are indicated, X is neither DR3 or DR4.

In addition to the six affected family members with diabetes and end-stage renal disease, eight additional family members were available for this study. Sanger sequencing confirmed the carrier status of the six affected family members and identified four additional carriers and four non-carriers of the *ADIPOQ* mutation in the family (Fig. 1a and d). Five of these carriers (Family Members III:3, III:8, IV:2, V:1, and V:2) had a diagnosis of diabetes either shortly before enrollment to the Joslin Study on the Genetics of Type 2 Diabetes^15^ or following their participation in this study; neither, likely due to their short duration of diabetes at enrollment and the lack of follow-up, developed diabetic kidney disease or end-stage renal disease to our knowledge. The two other carriers (Family Members III:2 and IV:1) were nondiabetic at enrollment and no follow-up data are available. Among carriers of the *ADIPOQ* mutation, only three possessed either one human leukocyte antigen (HLA) DR3 or DR4 risk allele, suggesting that their diabetes is likely not due to the autoimmune form of this disease (Table 1).

### Circulating adiponectin and ceramide levels of mutant ADIPOQ carriers and noncarriers

**Fig. 2:**
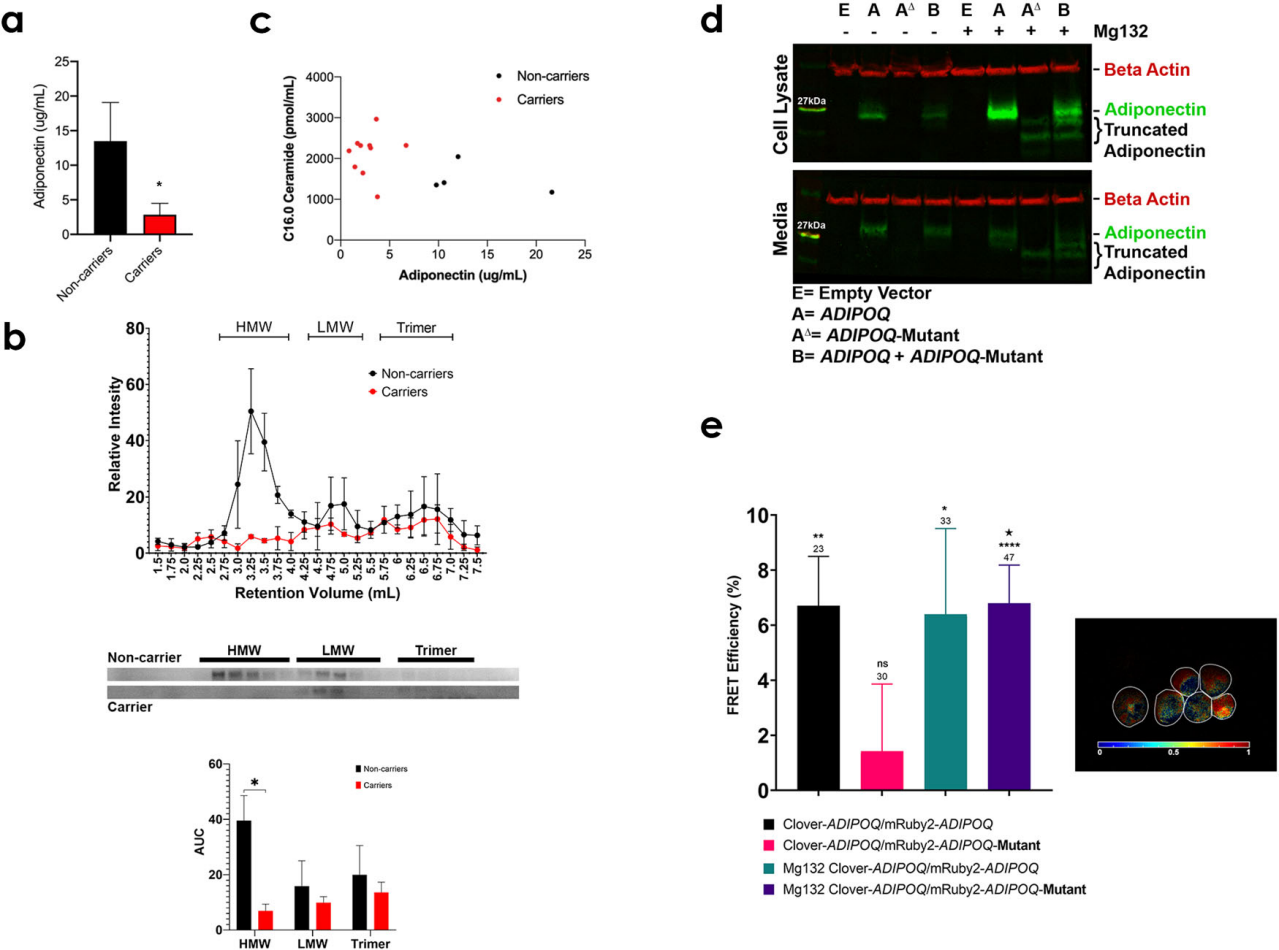
Circulating adiponectin and ceramide levels and characterization of the ADIPOQ mutation. **a** The level of circulating adiponectin, measured by enzyme-linked immunosorbent assay (ELISA), in noncarriers (black, *n* = 7) and carriers (red, *n* = 8) of the *ADIPOQ* mutation (two-tailed t-test, *α* = 0.05, *p value ≤ 0.05, error bars SEM). **b** The intensity values of bands from western blotting for noncarrier (black, *n* = 2) and carrier (red, *n* = 2) patients. Carriers of the *ADIPOQ* mutation lack bands in FPLC fractions associated with high molecular weight (HMW) adiponectin complexes (error bars SEM). Representative western blots are shown and area under the curve was calculated for each peak, HMW, low molecular weight (LMW), and adiponectin trimers (2-way ANOVA with Fisher’s LSD correction, *α* = 0.05, *p* = 0.018, error bars are SEM). **c** The levels of circulating adiponectin, measured by ELISA, in noncarriers (black, *n* = 4) and carriers (red, *n* = 10) of the *ADIPOQ* mutation relative to C16.0 ceramide levels, measured by LC-MS/MS. **d** Western blot showing wild-type and mutant adiponectin produced in HEK293T cells. The upper blot represents cell lysates and the lower blot shows the two proteins excreted into the cell media. The lanes denoted with a+ were samples taken from cells treated with 20 µg/mL of MG132. Beta actin was used as a loading control. **e** The FRET efficiency calculated from acceptor bleaching and calculating the increase of intensity of the donor fluorophore. Clover-ADIPOQ and mRuby2-ADIPOQ serve as a positive control for protein-protein binding and FRET. The Clover-ADIPOQ and mutant mRuby2-ADIPOQ cells show no significant FRET efficiency, though very little mutant mRuby2-ADIPOQ is observed in the cells. This is ameliorated by the addition of MG132 (20 µg/mL) blocking mutant mRuby2-ADIPOQ from being degraded. The number of cells counted for quantification is denoted above each bar in the graph. (Asterisks represent significance by a one-sample *t* test indicating that the chance of FRET occurring is above zero, *α* = 0.05, *p value ≤ 0.05, **p value ≤ 0.01, ****p value ≤ 0.0001. The large star represents a significant difference between non-treated and MG132 treated Clover-ADIPOQ and mutant mRuby2- ADIPOQ cells by two-tailed *t* test, *α* = 0.05, *p* = 0.04, error bars SEM). The panel also shows an intensity modulated ratiometric image of cells treated with MG132 that produce both Clover-ADIPOQ and the mutant mRuby2-ADIPOQ proteins. Red pseudo color shows pixels in which the donor intensity increases after photobleaching, indicating a FRET signal as the proteins interact.

To investigate whether the *ADIPOQ* mutation alters the level of circulating adiponectin, we measured adiponectin in serum from all 14 family members. Carriers of this truncating mutation had significantly reduced circulating adiponectin, less than 20% of the levels found in noncarriers (Fig. 2a). We also fractionated serum using fast protein liquid chromatography (FPLC)-based gel filtration and each fraction was probed for adiponectin by Western blot. Carriers of the mutation lacked high molecular weight (HMW) adiponectin complexes (Fig. 2b and Supplementary Fig. 5a, b), while HMW adiponectin was the most abundant isoform in noncarriers. As adiponectin’s role as a protective adipokine has been attributed to its ability to reduce lipotoxic ceramides^5^, we used liquid chromatography with tandem mass spectrometry (LC-MS/MS)^16^ to analyze ceramide level in carriers and noncarriers from this family. As anticipated, we observed that carriers of the *ADIPOQ* mutation had on average a 35% increase in C16.0 ceramide levels compared to noncarriers (Fig. 2c) and even greater increases relative to nondiabetic controls (Supplementary Fig. 6).

### Functional characterization of the ADIPOQ mutation

Previous studies have shown that missense point mutations in adiponectin impair multimerization, which is critical for formation of functional trimers, hexamers, and high molecular weight multimers^12,13^. As the *ADIPOQ* mutation significantly alters adiponectin structure, we hypothesized that this mutation likely disrupts adiponectin function. To investigate this, we developed gene expression constructs to allow tight expression of wild-type human adiponectin and the *ADIPOQ* mutant form of the protein within a single vector in human stable transfected embryonic kidney 293T (HEK293T) cells (Supplementary Fig. 7). Expression of mutant adiponectin can only be visualized when protein degradation is inhibited by MG132 (Fig. 2d and Supplementary Fig. 5c). Cells that express both the wild-type and the mutant protein express less adiponectin inside the cell, with reduced amounts secreted into the media. In the presence of MG132, the mutant protein can be visualized and cells producing both wild-type and mutant protein show a significant reduction of adiponectin within the cell and excreted to the media. To determine whether mutant and wild-type adiponectin interact, we conducted FRET assays using adiponectin tagged with either Clover or mRuby2 (Supplementary Fig. 8). As expected, acceptor-bleaching of living cells using confocal microscopy revealed that these adiponectin monomers interacted, forming multimers (Fig. 2e). When the mRuby2-tag was added to the mutant adiponectin construct, rather than the non-mutated isoform, FRET efficiency decreased substantially, likely due to degradation of the mutant form of the protein by the cells. The proteasome inhibitor MG132 increased FRET efficiency in these cells. The intensity modulated ratiometric image (Fig. 2e) represents MG132-treated cells that express both wild-type and mutant adiponectin. The sensitized emission model of FRET showed moderate levels of heterologous protein interaction that increased substantially following treatment with MG132 (Supplementary Fig. 8). Taken together, these data show that adiponectin and the truncated variant produced by the *ADIPOQ* mutation interact, leading to decreased stability of the wild-type adiponectin. It is in this way that the mutant adiponectin exerts a dominant-negative effect that results in significantly reduced adiponectin levels.

## Discussion

We report the identification of a rare truncating mutation in *ADIPOQ* in a multigenerational family enriched for diabetes and end-stage renal disease. Although we cannot rule out that other renal pathologies account for the kidney disease observed in this family, as no other monogenic kidney disease gene (e.g., *PKD1*, *PKD2*, *COL4A3/4/5*, etc.) emerged from our genetic analysis of this family, suggesting that other heritable forms of kidney disease are unlikely, diabetes is the suspected cause. Our data show that this mutation results in dramatically reduced circulating adiponectin (<20% of normal levels) and high ceramide levels (more than a 30% increase in C16.0 ceramides compared to noncarriers). The *ADIPOQ* mutation, located in exon 3 (i.e., the terminal exon) of the encoded protein, causes a frameshift in the sequence encoding the collagenous domain of adiponectin and disrupts formation of its globular domain. Given its location, we hypothesize that the resulting mutant protein escapes the usual mechanism of nonsense-mediated decay and is able to form characteristic multimers, but targets them for degradation. The resultant reduction in functional adiponectin multimers leads to increased ceramide levels. The low-adiponectin, high ceramide state in this family is associated with diabetes and progressive loss of kidney function.

Rare missense mutations, primarily at conserved amino acid residues in adiponectin’s collagenous and globular domains were previously identified in individuals with type 2 diabetes and hypoadiponectinemia^12,13,17–19^. Interestingly, similar to carriers of the *ADIPOQ* mutation identified in our study, several point mutations, within the globular domain of adiponectin, cause low circulating levels of adiponectin^18^. Most notably, adiponectin p.R112C and adiponectin p.I164T show 27% and 32% of the circulating adiponectin seen in wild-type adiponectin carriers, respectively. In this study, *ADIPOQ* mutation carriers show 24% of the circulating adiponectin of unaffected family members. Each mutant adiponectin similarly disturbs multimer assembly and secretion of the wild-type protein. Unlike the truncated adiponectin studied here, which is nearly undetectable in the absence of MG132, these previously identified point mutants form stable monomers with levels of intracellular protein expression similar to wild-type adiponectin. A portion of adiponectin is known to remain within the adipocyte, and can maintain potent ceramide-lowering biological activity.

Expanding on these findings, in addition to reporting the first strongly pathogenic mutation in *ADIPOQ* that leads to familial hypoadiponectinemia, we also demonstrate that this mutation results in a prominent increase in C16.0 ceramide levels. Although it remains unclear why C16 ceramides are more pathological than other ceramide species, recent studies have demonstrated that the Cers6 enzyme that generates C16 ceramides is selectively upregulated in obese humans and mice^20^ and these ceramides uniquely trigger mitochondrial dysfunction^21^. Conversely, we have linked adiponectin and adiponectin receptors AdipoR1 and AdipoR2 to ceramide catabolism and C16 ceramides are uniquely altered by overexpression of adiponectin, AdipoR1, or AdipoR2 in preclinical studies^22^. Thus, higher C16 ceramides associated with the *ADIPOQ* mutation are likely a result of diminished ceramidase activity and ceramide catabolism resulting from the inability of reduced functional adiponectin multimers to stimulate its two receptors, ADIPOR1 and ADIPOR2^5^. In the total absence of adiponectin, these receptors fail to catalyze ceramide degradation^6^. Importantly, ceramides, a class of deleterious sphingolipids, have previously been shown to contribute to cellular dysfunction that causes impaired insulin action, induces β cell death, and exacerbates kidney failure^23^.

Several recent studies have demonstrated associations between abnormal long-chain ceramides, as well as other sphingolipids species in this pathway, and albuminuria, estimated glomerular filtration rate decline, and end-stage renal disease in subjects with diabetes^24–26^. Further supporting the role of ceramide biosynthesis in diabetic kidney disease, large-scale genetic studies have linked a common deleterious missense variant in ceramide synthase-2 (*CERS2*), a ceramide-synthesizing gene in the sphingolipid biosynthesis pathway, with reduced renal function and diabetic kidney disease risk^27,28^. While the heritability of ceramide levels has been estimated to be as high as 0.52, our understanding of the genetic factors that underlie variation of ceramide levels is woefully incomplete^29^. Indeed, variability in ceramide levels, even among carriers of the *ADIPOQ* mutation, suggest that this is likely a polygenic trait influenced by a network of genetic, as well as environmental, factors. Of note, patient III:2, successfully avoided diabetes and is the only carrier of normal body weight. Conversely, many noncarriers in this study were also diabetic. Maternal-fetal environment^30^, shared dietary patterns amongst family members, and epigenetic alterations^31^ could contribute to some degree of diabetes risk. Notably, adiponectin null mice even produce breastmilk that is proinflammatory, which can alter the health status of their offspring^32^. Although ceramides have clearly been linked to diabetic kidney disease, the underlying factors that lead to ceramide biology dysfunction are largely unknown, highlighting a need for additional research in this area.

In summary, we identified the first multigenerational family with a protein truncating mutation in *ADIPOQ*, diabetes, and end-stage renal disease. This mutation impairs adiponectin multimer formation, dampens intracellular protein expression, and impacts adiponectin’s role in glucose and lipid metabolism. Our findings further implicate adiponectin dysfunction in diabetes and diabetic kidney disease and link abnormalites in circulating adiponectin with elevated ceramide levels through impaired activation of its cognate receptors. Importantly, compounds exist (e.g., AdipoRon^33^) that activate adiponectin receptors and lower ceramide abundance. For this family, adiponectin mimetics represent a potential personalized medicine approach to counteract deficient adiponectin levels and increased ceramide levels due to this mutation. More broadly, interventions to lower ceramides could prove to be novel therapies for treating diabetes or slowing the progression of diabetic kidney disease.

## Methods

### Family enriched for diabetes and end-stage renal disease

Individuals from the family examined in this study were enrolled in the Joslin Study on the Genetics of Type 2 Diabetes between 1993 and 2003^15^. Briefly, between 1993 and 2003, families with an apparent autosomal dominant mode of inheritance of type 2 diabetes (T2D), irrespective of their nephropathy status, were recruited to the Joslin Study of Genetics of Nephropathy in Type 2 Diabetes Family Collection through T2D probands receiving medical care at the Joslin Clinic. The protocols and written informed consent procedures used in this study were approved by the Committee on Human Subjects of the Joslin Diabetes Center.

All participating family members provided written informed consent prior to participating in this study. All family members were of European ancestry. After obtaining informed written consent, trained recruiters administered previously described study protocols that included a structured interview, seated blood pressure measurements, and the collections of blood and urine samples. All blood and urine specimens have been stored at −80 °C since the time of their collection. ESRD status for members of this family was updated through the United States Renal Data System.

### Genetic analyses

Whole genome sequencing of DNA samples from six individuals with ESRD was performed on the Illumina HiSeq2500 platform available at Macrogen (Rockville, MD). FASTQ files from paired-end sequencing were subsequently aligned to the Hg19/GRCh37 reference genome using the Sentieon Genomics DNASeq software pipeline per the developer’s guidelines^34^. The DNASeq pipeline incorporates the Broad Institute’s BWA-GATK Best Practices Workflow but performs alignments and variant calling in a computationally efficient process. The six individual samples were jointly genotyped with 524 ethnically matched background controls comprised of 291 individuals from the 1000 Genomes Project and 233 healthy samples from the Utah Genome Project^35^. The pipeline created individual genomic variant call files and a final jointly-called variant call file (VCF). Sample qualities in final VCF were evaluated using Peddy^36^ to determine and confirm sex, relatedness, heterozygosity and ancestry of each individual to identify any potential sample quality issues. The VCF was functionally annotated using ANNOVAR for downstream analyses^37^.

A unified linkage analysis and rare variant association testing using pedigree Variant Annotation, Analysis, and Search Tool (pVAAST), version 2.2.0, was performed on six individual sample target genomes and 524 ethnically matched background controls^35^. pVAAST incorporates pedigree information into disease-gene prioritization procedures that incorporates amino acid substitution and calculates burden of each gene candidate. pVAAST was performed using 1 × 10^−7^ permutations and configured to evaluate penetrance values in the range of 0.5–0.995, with LOD and CLRT filters removed. In keeping with best practices from the developers of this tool, we considered any gene with a variant achieving a pVAAST *p* value < 0.005 as a potential positive signal. Additional reranking of pVAAST outputs occurred using Phenotype Driven Variant Ontological Re-ranking tool (PHEVOR) using Human Phenotype Ontology (HPO) terms and connections to Gene Ontology (GO) terms to prioritize potentially damaging alleles, using the HPO terms a) for kidney disease: HP:0000077 (Abnormality of the kidney), HP:0000112 (Nephropathy), and HP:0003774 (Stage 5 chronic kidney disease), and b) for diabetes: HP:0000819 (Diabetes) and HP:0005978 (Type 2 diabetes mellitus) and a combination of the pVAAST *p* value and Phevor score^38^.

Sanger sequencing confirmation of the *ADIPOQ* variant was performed in all 6 individuals with ESRD in this family as well as 8 additional family members for whom DNA was available. The genomic region covering the variant was PCR amplified using the Qiagen *Taq* PCR amplification kit (Qiagen, Valencia, CA) per the manufacturer’s protocol and the following primer pair: forward *5*′*–GGCTGTAACCAACCTAGGCAGG–3*′ and reverse *5*′*–AGGCAAAGTAGTACAGCCCAGG–3*′. Sanger sequencing was performed using BigDye Terminator v3.1 Cycle Sequencing chemistry (Life Technologies, Carlsbad, CA). Capillary electrophoresis was performed by the DNA Sequencing Core Facility at the Health Science Center at the University of Utah using a 3730 DNA Analyzer (Applied Biosystems, Foster City, CA). The resulting chromatograms were analyzed using Sequencher version 5.4.6 (Gene Codes Corporation, Ann Arbor, MI).

All 14 family members were genotyped on the HumanCore BeadChip (Illumina, San Diego, CA, USA), which contains 250,000 genome-wide tag SNPs (and other variants) and over 200,000 exome-focused variants, by the DNA Sequencing Core Facility at the Health Science Center at the University of Utah. All samples were passed through a stringent quality control protocol that included filtering for low-quality variants (e.g., call rates <95% and excessive deviation from Hardy–Weinberg equilibrium).

HLA alleles DR3 (haplotype DQA1*05:01-DQB1*02:01) and DR4 (haplotype DQA1*03-DQB1*03:02) were predicted for all 14 family members using genome-wide genotyping data, the SNP2HLA software^39^, and genome-wide genotyping data from the HLA-region and the Type 1 Diabetes Genome Consortium’s reference genotype-HLA-allele panel.

### Circulating adiponectin

Circulating adiponectin levels were measured in serum from 14 family members (including 4 noncarriers and 10 carriers of the *ADIPOQ* mutation) using an enzyme-linked immunosorbent assay (ELISA) (Millipore EZHADP-61K). Protein concentration was assessed using a BCA kit (Pierce) prior to load equivalent protein levels from all patients. The ELISA was measured using a ThermoFisher Varioskan Lux Microplate Reader.

### Fast protein liquid chromatography (FPLC)

FPLC was performed as previously described^40^. Serum (50 µL) from two representative noncarrier or carrier siblings was injected into an ÄKTA Go FPLC (GE Healthcare). A Superdex 200 10/300 GL column (GE Healthcare) was used to separate adiponectin complexes in HEPES/Ca^2+^ buffer (25 mM HEPES; 150 mM NaCl; and 1 mM CaCl_2_, pH 7.4). 250 µL fractions were collected over a 20 mL retention volume. The retention volumes found to contain adiponectin were then used to run Western blots to determine HMW, LMW and trimeric adiponectin. Samples were run on an SDS-Page gel (BioRad Criterion TGX) after being reduced in Laemmli and 355 mM 2-mercaptoethanol and boiled for 10 min. The gel was transferred to PVDF membrane (BioRad). The membranes for each patient were blocked in 5% BSA then probed for adiponectin overnight with rabbit polyclonal anti-adiponectin at a 1:1000 dilution (Abcam, ab75989). There were washed then stained with goat-anti-rabbit AlexaFluor Plus 680 (Invitrogen) at a 1:4000 dilution. Then washed again and imaged on a ThermoFisher iBright system. Western blots derive from the same experiment and were processed in parallel.

### Lipidomic analysis

Ceramide levels were measured using targeted lipidomic profiling and liquid chromatography with tandem mass spectrometry (LC-MS/MS) at the University of Utah’s Metabolomics Core. Analysis of lipid species, including C16.0 ceramides, was performed as previously been described^16^. Briefly, these determinations were obtained from serum specimens from 14 family members as well as 25 unrelated nondiabetic controls using targeted LC-MS/MS platforms at the University of Utah’s Metabolomics Core with each lipid normalized to an internal standard using an established and previously described method developed by the Summers lab^16^. Statistical analysis was performed using Student’s *t* test in SAS for Windows, version 9.2 (SAS Institute, Cary, NC) following log10 transformation of these data and results are considered significant at the *P* < 0.05 level.

### Gene expression vectors

Gene sequences for human wild-type and mutant *ADIPOQ* were cloned into the pVitro2 vector (InvivoGen) expressing blasticidin resistance (Fig. S6). The vector is designed for dual gene expression with both genes driven by two human ferritin composite promoters. Following the addition of wild-type *ADIPOQ* is an IRES followed by the *bsr* gene allowing for blasticidin resistance. For cells that express only wild-type or mutant *ADIPOQ*, the gene sequences were disrupted with a premature stop codon so that only one variant of adiponectin is produced. The vectors were transfected into HEK293T cells (ATCC, CRL-11268) using JetPrime (Genesee Scientific). Twenty-four hours after transfection, 20 µg/mL blasticiden was added to the cell culture media. The cells were grown and split three times to ensure stable expression of the genes. The cells were maintained in 10 cm cell culture dishes in DMEM with 10% FBS, pen/strep, and blasticidin. For cells treated with the proteasome inhibitor MG132 (Sigma), healthy cells were treated for 12–18 h with a concentration of 20 µM in FBS free media.

### Western blotting

Stable expressing cells were grown to 80–90% confluency. Then media was removed and supplemented with DMEM with no FBS. Twelve to eighteen hours later the media was collected with the addition of protease inhibitors (Roche) and put-on ice. The cells were lysed using 200 µL of ice cold radioimmunoprecipitation assay buffer containing protease inhibitors (Roche). The cells were vortexed and lightly sonicated to form a homogeneous solution. Protein concentration was assessed using a BCA kit (Pierce). For traditional Western blotting, 2 µg of protein was reduced using Bolt Reducing Agents (ThermoFisher) and heated for 5 min. Protein was run on Invitrogen Bolt 4–12% Bis-Tris gels (ThermoFisher) and then transferred to a nitrocellulose membrane using the Invitrogen Power Blotter. The membranes were then processed for immunodetection using the iBind Western System. Primary antibodies were selected to target adiponectin (Rabbit polyclonal anti-adiponectin from Abcam, ab75989) and beta-actin as a loading control (Mouse monoclonal anti-beta-actin from Cell Signaling, 3700S). Both were used at a 1:1000 dilution. The adiponectin antibody selected for use was made using a peptide that falls within both the wild-type and mutant adiponectin protein. Antibodies targeting the N-terminus and the C-terminus of adiponectin were also used to confirm that the mutant protein is not detected when using a C-terminus antibody (data not shown). The secondary antibodies used were Alexa-fluor Plus 488 Anti-Mouse (ThermoFisher, A32723) and Alexa-fluor Plus 800 Anti-Rabbit (ThermoFisher, A32735) at a 1:2000 dilution. The blots were imaged on a ThermoFisher iBright system. All blots derive from the same experiment and were processed in parallel.

### FRET constructs and analysis

To assess if wild-type adiponectin and the mutant adiponectin protein could interact, we designed constructs with each protein tagged with a FRET sensor (Fig. S7). We chose a green fluorescent protein derivative called Clover and a red fluorescent protein derivative called mRuby2 to act as our donor/acceptor pair. Both Clover and mRuby2 were designed and proven to be excellent FRET sensors^41^. Either Clover or mRuby2 were attached to the protein via a linker sequence at the N-termina (since the same C-terminus is not present in the mutant protein). Individual wild-type adiponectin or mutant adiponectin were labeled with Clover or mRuby2 as single expressing constructs to gather spectral data from individual fluorophores. A double expressing construct with wild-type adiponectin labeled with Clover and wild-type adiponectin labeled with mRuby served as a FRET positive control since it is known that adiponectin forms multimers with itself. A double expressing construct with wild-type adiponectin labeled with Clover and mutant adiponectin labeled with mRuby2 served as the experimental group.

FRET readings were recorded via two methods, acceptor bleaching and sensitized emission.

### Acceptor bleaching model

In this model, the acceptor fluorophore is photobleached and donor fluorophore intensities are taken prior and after the photobleaching has occurred. If FRET is occurring, the energy that would have gone toward exciting the acceptor remains with the donor, meaning that the donor intensity is increased after photobleaching the acceptor. The stable expressing cells were grown in coverslip bottom chamber slides using FluoroBrite DMEM (Gibson) with 2% FBS. The slides were mounted into a Pathology Devices, Inc stage heater kept at 37 °C and imaged on a Nikon A1R confocal microscope with a 40× oil immersion objective. Cells were quickly identified and focused using epifluorescence. A pre-bleach image was obtained using a 488 nm argon gas laser to excite Clover and a 561 nm sapphire diode laser to excite mRuby2 using predetermined laser settings for adequate imaging. After a pre-bleach image was obtained the 488 nm laser was turned off and the 561 nm laser was set to 100% power and allowed to excite the cells for 10 min to bleach the mRuby2 signal. Afterwards, a post-bleach image was acquired using the same laser and acquisition settings as the pre-bleach image. Cells containing the empty vector that produce neither Clover nor mRuby2 were imaged to provide background intensities of the cells. The intensity for Clover and mRuby2 in each cell was measured in FIJI imaging software in both the pre- and post-bleach images. The average background intensity from non-expressing cells was subtracted from the intensity measurements of the pre- and post-bleach images. Only cells that had a ≥10% reduction in mRuby2 intensity were used for quantification. FRET efficiency was calculated by $1 - \frac{{F_{da}}}{{F_d}}$, where *F*_da_ is the intensity of the donor fluorophore before bleaching and *F*_d_ is the intensity of the donor fluorophore after the 10-min bleach. The FRET efficiency percent was graphed using Prism. Intensity modulated ratiometric images were created using RatioImage in MatLab.

### Sensitized emission model

In this model, the intensity of the acceptor fluorophore is measured to determine if it gains intensity when the donor fluorophore is physically close. The stable expressing cells were grown in a Greiner µClear bottom black-walled 96-well plate using DMEM with 10% FBS, pen/strep, and blasticidin ensuring that each FRET construct (individual expressing cells and dual Clover/mRuby2 expressing cells) were in triplicate. Once the cells reached 70–80% confluency, the media was replaced with FluoroBrite DMEM (Gibson) with no FBS. The cells were treated with MG132 at this time and incubated for 12–18 h. The plate was then analyzed using a ThermoFisher Varioskan Lux Microplate Reader with the chamber heated to 37 °C and maintaining 5% CO_2_. The Varioskan was set to read 29 readings from the bottom of each well. To detect Clover, intensity excitation was set to 505 nm and emission detection was at 515 nm. To detect mRuby2, intensity excitation was set to 559 nm and emission detection was at 600 nm. FRET readings for dual Clover/mRuby2 expressing cells were taken with excitation set at 505 nm, to excite Clover and emission detection was set at 600 nm to read mRuby2 fluorescence. Spectral data with each excitation setting were also obtained to verify that the fluorophores emitted at their ideal peak emission curve (data not shown). Fluorescent readings were background subtracted from readings from cells with the empty vector. This FRET method requires subtracting out the spectral bleed-through derived from the donor emission spectrum overlap with the acceptor and the direct excitation of the acceptor by the donor’s excitation light to verify actual FRET. The corrected FRET ratio (*F*_C_) was obtained by:

$$F_C = \frac{{Raw\,FRET\,intensity - DSBT}}{{Acceptor\,intensity - ASBT}},$$

where DSBT is the donor spectral bleed-through and the value obtained from the wild-type adiponectin tagged with Clover in the 505–600 nm reading. ASBT is the acceptor spectral bleed-through and is obtained from the mutant adiponectin tagged with mRuby2 in the 505–600 nm reading. Acceptor intensity is the measure from the 559 nm excitation and 600 nm emission reading. The corrected FRET ratio was graphed using Prism. The experiment was repeated two other times with similar results.

### Reporting summary

Further information on research design is available in the Nature Research Reporting Summary linked to this article.

## Supplementary information

Supplemental Information_Clean

Reporting Summary

## Data Availability

The data that support the findings of this study are available from the corresponding author upon request. All sequencing data from this study are publicly available from dbGaP (accession phs002959.v1).
