# Supplementary material for: A dominant negative ADIPOQ mutation in a diabetic family with renal disease, hypoadiponectinemia, and hyperceramidemia
Source: NPJ Genom Med. 2022 Jul 22;7:43. doi: 10.1038/s41525-022-00314-z (PMC9307825; doi:10.1038/s41525-022-00314-z)
Supplement: Supplementary file 1 — Supplemental Information_Clean [file 41525_2022_314_MOESM1_ESM.docx]

**A Dominant Negative *ADIPOQ* Mutation in a Diabetic Family with Renal Disease, Hypoadiponectinemia, and Hyperceramidemia**

**Supplementary Information**

Christopher A. Simeone,^1,2*^ Joseph L. Wilkerson,^3*^ Annelise M. Poss,^3^ James A. Banks,^3^ Joseph V. Varre,^3^ Jose Lazaro Guevara,^1,2^ Edgar Javier Hernandez,^1,4^ Bushra Gorsi,^1,4^ Donald L. Atkinson,^3^ Tursun Turapov,^3^ Scott G. Frodsham,^2^ Julio C. Fierro Morales,^2^ Kristina O’Neil,^5^ Barry Moore,^1,4^ Mark Yandell,^1,4^ Scott A. Summers,^3^ Andrzej S. Krolewski,^5,6*^ William L. Holland,^3*^ Marcus G. Pezzolesi,^1,2,7*^

^1^Department of Human Genetics, University of Utah School of Medicine, Salt Lake City, Utah, 84112

^2^Division of Nephrology and Hypertension, Department of Internal Medicine, University of Utah School of Medicine, Salt Lake City, Utah, 84132

^3^Department of Nutrition and Integrative Physiology, University of Utah College of Health, Salt Lake City, Utah, 84112

^4^Utah Center for Genetic Discovery, Department of Human Genetics, University of Utah School of Medicine, Salt Lake City, Utah, 84112

^5^Section on Genetics and Epidemiology, Research Division, Joslin Diabetes Center, Boston, Massachusetts, 02115

^6^Department of Medicine, Harvard Medical School, Boston, Massachusetts, 02115

^7^Diabetes and Metabolism Research Center, University of Utah School of Medicine, Salt Lake City, Utah, 84108

*Authors Contributed Equally

**Table of Contents**

Supplementary Figure 1. pVAAST Analysis of Six Family Members with Diabetes and End-stage Renal Disease. 3

Supplementary Figure 2. PHEVOR Plot using Human Phenotype Ontology (HPO) Terms for Diabetes: HP:0000819 (Diabetes) and HP:0005978 (Type 2 diabetes mellitus). 4

Supplementary Figure 3. PHEVOR Plot using HPO Terms for Kidney Disease: HP:0000077 (Abnormality of the kidney), HP:0000112 (Nephropathy), and HP:0003774 (Stage 5 chronic kidney disease). 5

Supplementary Figure 4. Alignment of Wildtype and Mutant Adiponectin Protein Sequences. 6

Supplementary Figure 5: Fast Protein Liquid Chromatography (FPLC) and Western Blots for Wildtype and Mutant Adiponectin 7

Supplementary Figure 6. C16.0 Ceramide Levels are Elevated in *ADIPOQ* Mutation Carriers. 8

Supplementary Figure 7. Single vector to induce expression of wild-type and mutant *ADIPOQ* in HEK293T cells. 9

Supplementary Figure 8. Schematic of wild-type and mutant FRET tagged ADIPOQ 10

Supplementary Table 1. Top Ranked Genes from pVAAST Analysis. 12

Supplementary Table 2. Top Ranked Genes from PHEVOR Analysis (HPO terms: HP:0000819 (Diabetes), HP:0005978 (Type 2 diabetes mellitus), HP:0000077 (Abnormality of the kidney), HP:0003774 (Stage 5 chronic kidney disease), and HP:0000112 (Nephropathy)). 13

Supplementary Table 3. Top Ranked Genes from PHEVOR Analysis (HPO terms: HP:0000819 (Diabetes) and HP:0005978 (Type 2 diabetes mellitus)). 14

Supplementary Table 4. Top Ranked Genes from PHEVOR Analysis (HPO terms: HP:0000077 (Abnormality of the kidney), HP:0003774 (Stage 5 chronic kidney disease), and HP:0000112 (Nephropathy)). 15

**Supplementary Figure 1**

**Supplementary Figure 1. pVAAST Analysis of Six Family Members with Type 2 Diabetes and End-stage Renal Disease.** pVAAST was used to perform a unified linkage analysis and rare variant association testing using 6 individual sample target genomes and 524 ethnically matched background controls (291 1,000 Genomes and 233 Utah Genome Project healthy samples). -log10 p-values (Y-axis) from this analysis are plotted across the genome (X-axis).

**Supplementary Figure 2**

******

**Supplementary Figure 2. PHEVOR Plot using Human Phenotype Ontology (HPO) Terms for Diabetes: HP:0000819 (Diabetes) and HP:0005978 (Type 2 diabetes mellitus).** PHEVOR scores (y-axis) for each gene (dot) are plotted across the genome (x-axis, chromosomes 1-Y).

**Supplementary Figure 3**

**Supplementary Figure 3. PHEVOR Plot using HPO Terms for Kidney Disease: HP:0000077 (Abnormality of the kidney), HP:0000112 (Nephropathy), and HP:0003774 (Stage 5 chronic kidney disease).** PHEVOR scores (y-axis) for each gene (dot) are plotted across the genome (x-axis, chromosomes 1-Y).

**Supplementary Figure 4**


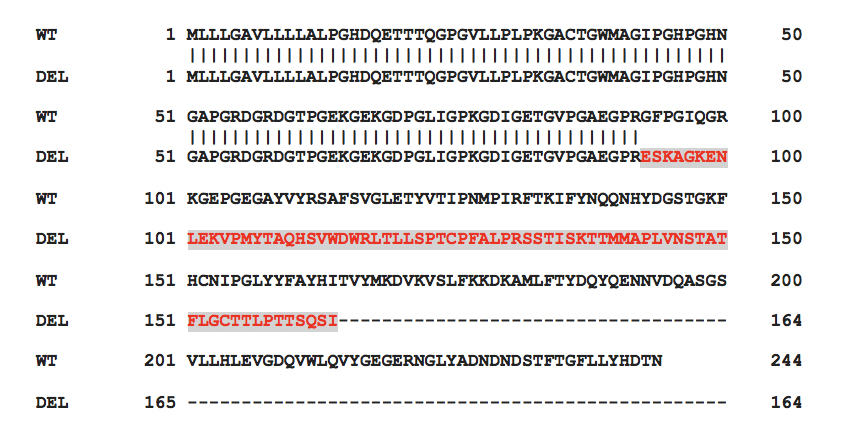


**Supplementary Figure 4. Alignment of Wild-type and Mutant Adiponectin Protein Sequences.** Sequence in red shows the consequence of the 10-nucleotide deletion which results in a frameshift at amino acid 93, generating a novel peptide (sequence in red) that terminates 73 amino acids after the deletion.

**Supplementary Figure 5**


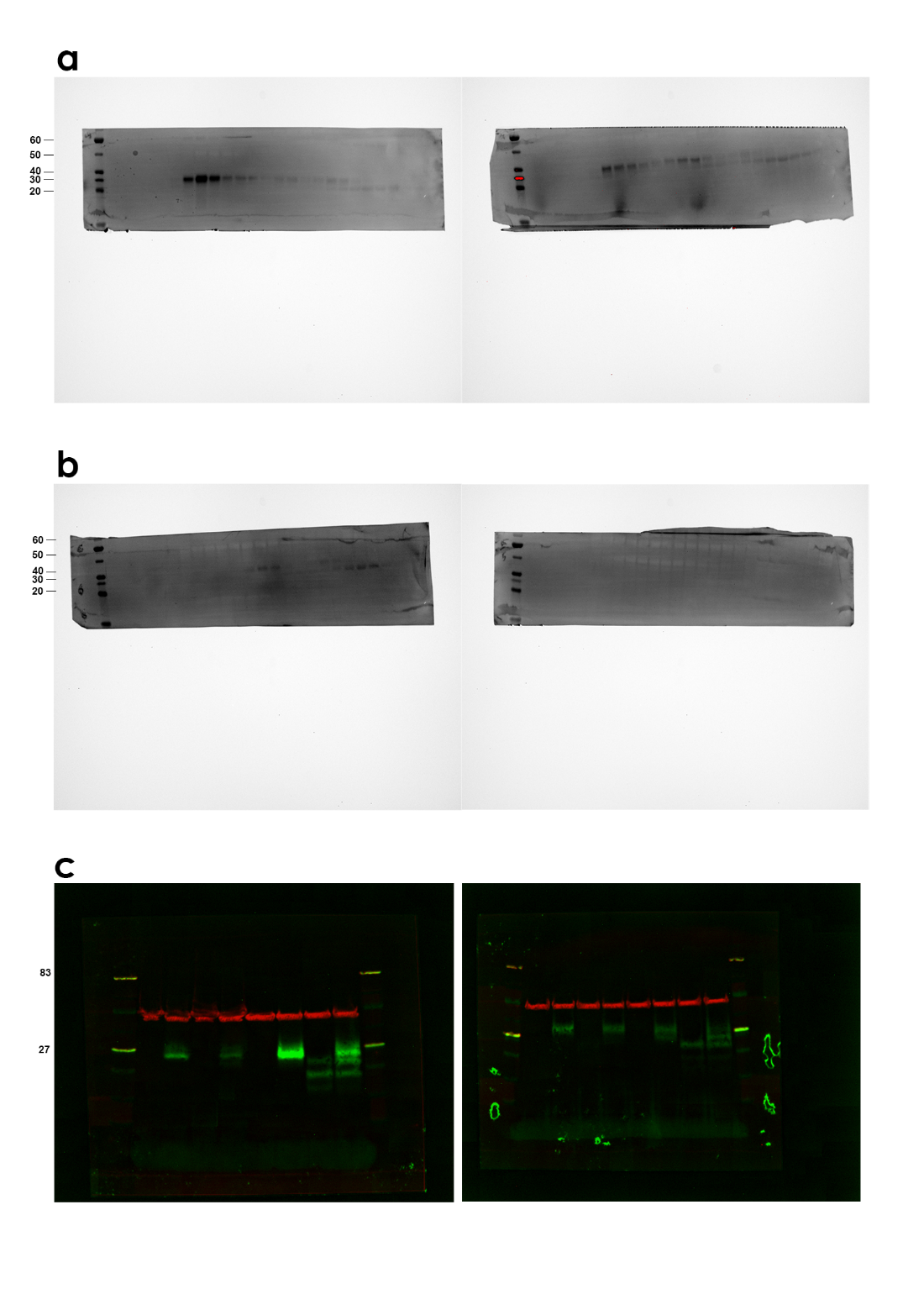


**Supplementary Figure 5. Fast Protein Liquid Chromatography (FPLC) and Western Blots for Wildtype and Mutant Adiponectin.** Adiponectin probed from A) two different wildtype carrier serum samples and B) two different mutant carrier serum samples after FPLC fractionation. C) Western blots of wildtype and mutant adiponectin from HEK293T cell lysate (left) and cell culture media (right). All images are unaltered and blots derive from the same experiment and processed in parallel. Beta actin was used as a loading control.

**Supplementary Figure 6**

**
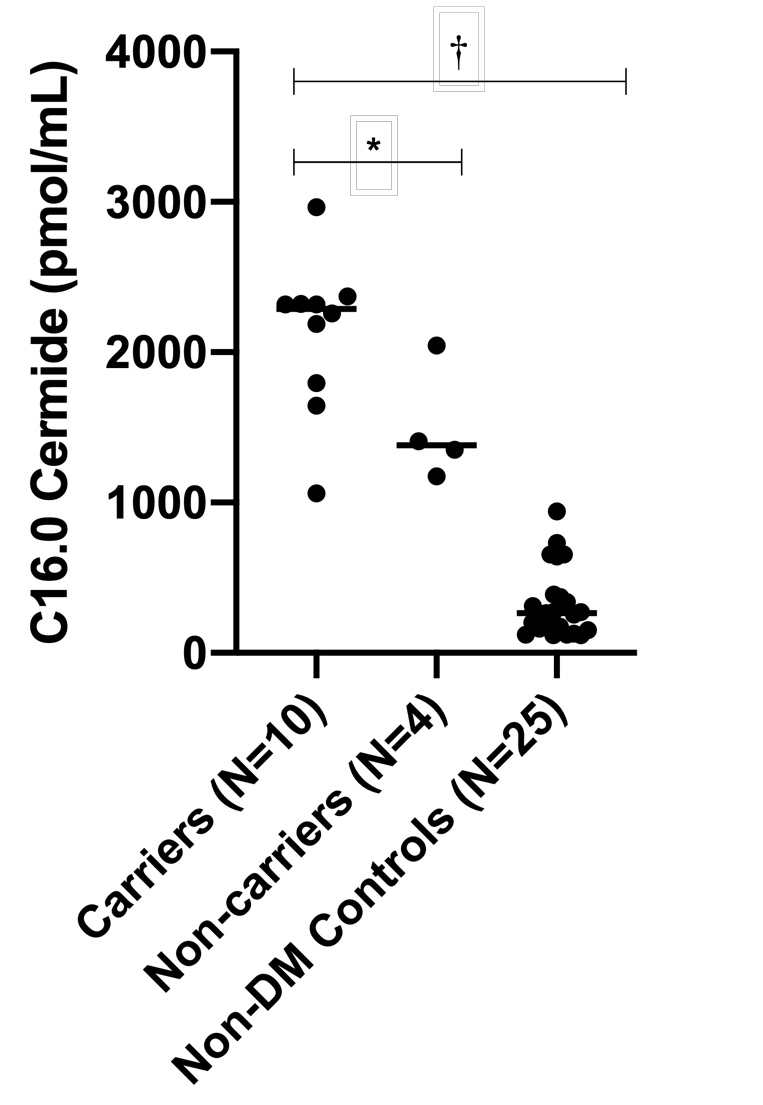
**

**Supplementary Figure 6.**  **C16.0 Ceramide Levels are Elevated in *ADIPOQ* Mutation Carriers.** Serum specimens from 14 family members with diabetes (10 carriers and 4 non-carriers) as well as 25 unrelated non-diabetic controls was analyzed by LC-MS/MS for ceramide levels. C16.0 ceramide levels are highly elevated in carriers of this mutation relative to non-carriers (*; p-value = 0.037) and compared to diabetic patients that do not carry the mutation (†; p-value < 0.001, two-tailed t-test, α=0.05).

**Supplementary Figure
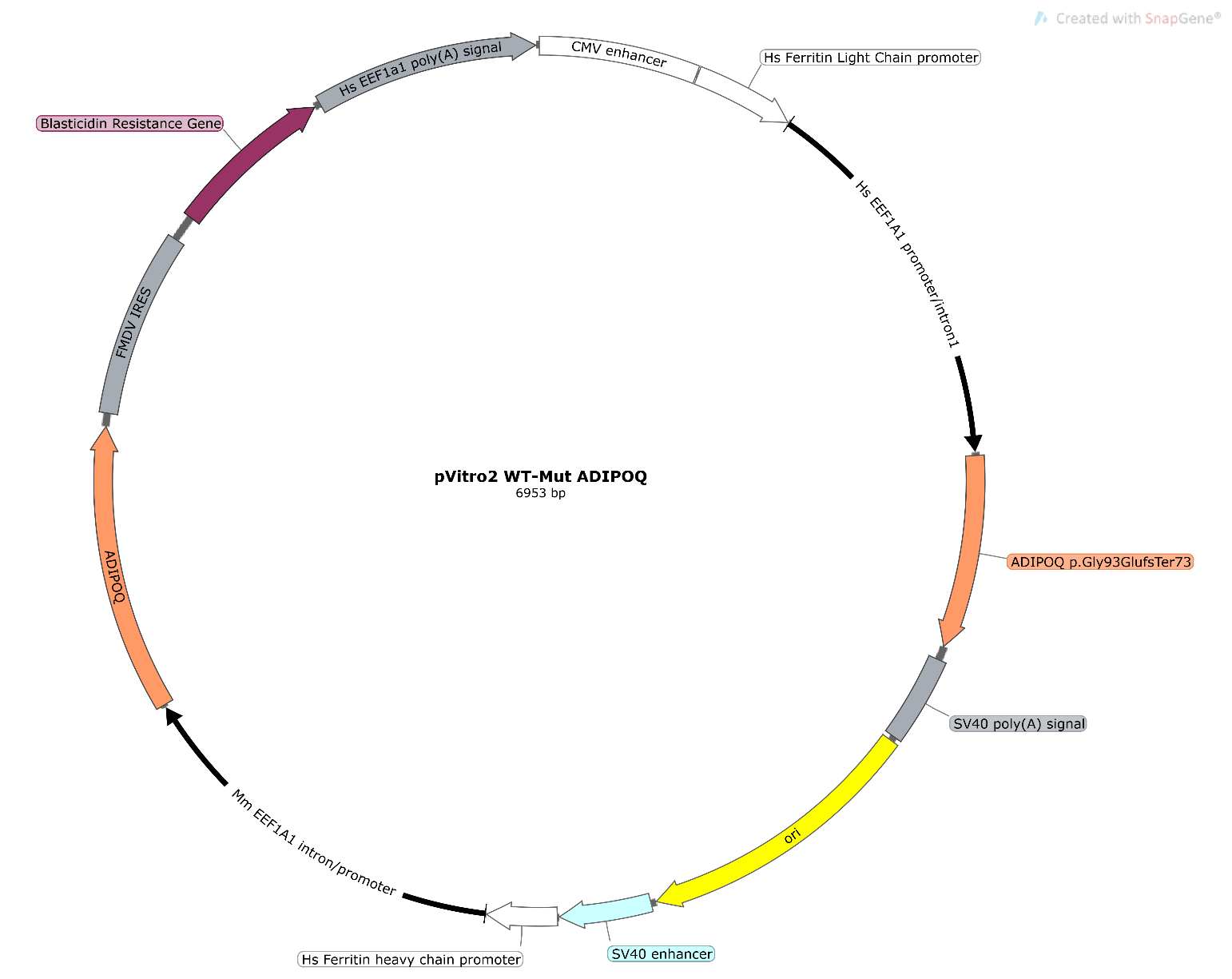
7**

**Supplementary Figure 7. Single vector to induce expression of wild-type and mutant *ADIPOQ* in HEK293T cells.** The pVitro2 vector was used for dual expression of adiponectin and the mutant variant. This vector allows for the production of both genes using the ferritin light and heavy chain promotors followed by EEF1A1 mammalian promotors. The vector is made selectable by adding an IRES followed by the blasticidin resistance gene after the transcription of *ADIPOQ*, which allows for tight regulation of protein production in the presence of blasticidin (20µM).

**Supplementary Figure 8**

**
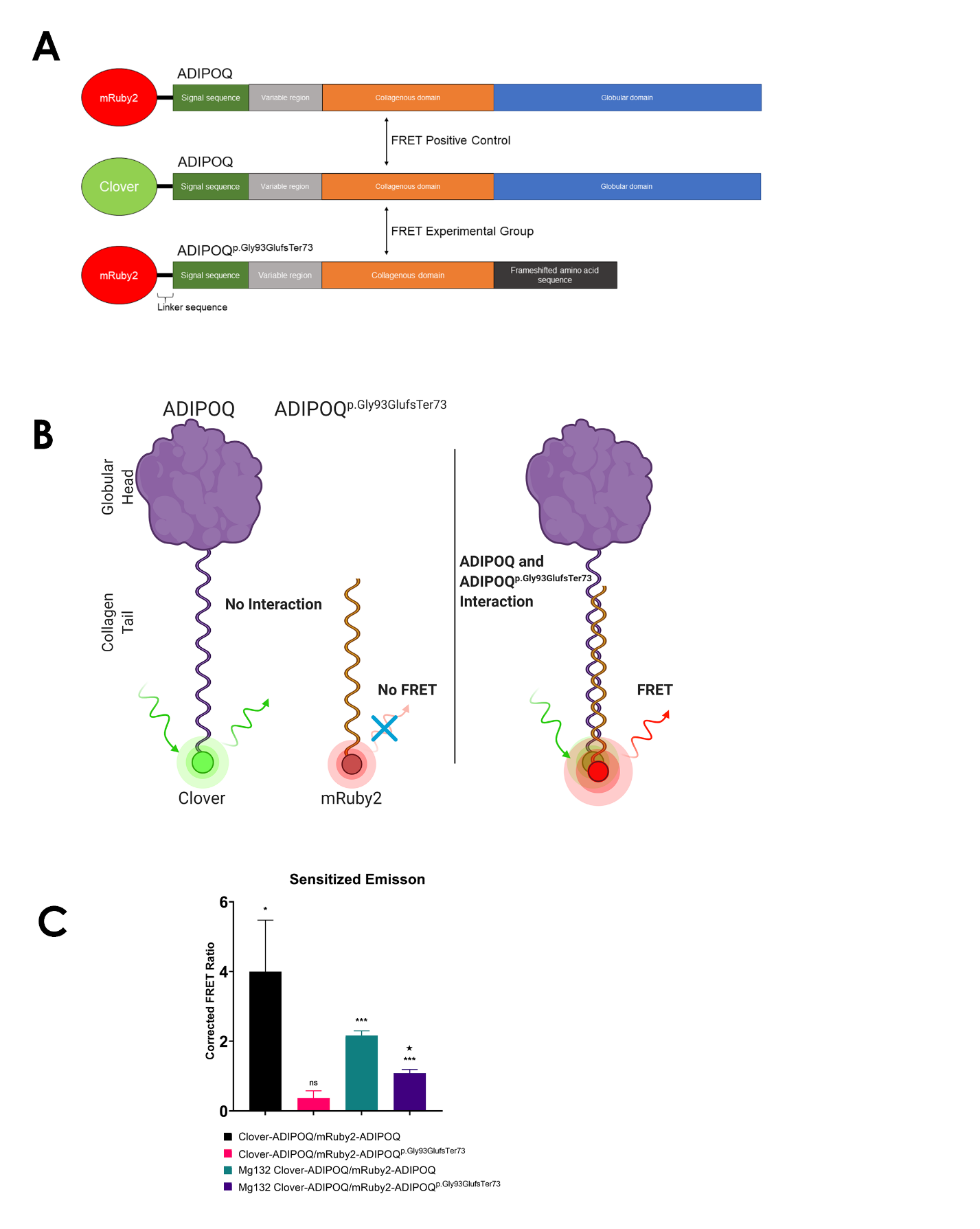
**

**Supplementary Figure 8. Schematic of FRET tagged wild-type and mutant ADIPOQ.** A. *ADIPOQ* was tagged with the FRET sensor Clover at the N-terminus. To serve as a positive control, *ADIPOQ* was also tagged with mRuby2 and expressed in the same vector as the Clover-*ADIPOQ.* To determine protein interaction with mutant adiponectin, mutant *ADIPOQ* was tagged with mRuby2 at the N-terminus and then expressed in the same vector as Clover-*ADIPOQ.* B. Schematic of FRET interaction between wild-type and mutant ADIPOQ (Created in BioRender). C. Panel E shows corrected FRET ratios from cells using sensitized emission readings generated using a fluorescent plate reader. As with the acceptor bleaching model, Clover-ADIPOQ and mutant mRuby2-ADIPOQ cells show no significant FRET occurring, and the addition of MG132 increased the FRET Ratio. (Asterisks represent significance by a one-sample t-test indicating that the chance of FRET occurring is above zero, n=6, α=0.05, *p-value≤0.05, ***p-value≤0.001, ****p-value≤0.0001. The large star represents a significant difference between non-treated and MG132 treated Clover-ADIPOQ and mutant mRuby2- ADIPOQ cells by two-tailed t-test, α=0.05, p=0.02, error bars are SEM.)

**Supplementary Table 1. Top Ranked Genes from pVAAST Analysis.**

| **Rank** | **Gene** | **P-value** | **P-value CI** | **Score** | **LOD** | **Variant** |
| --- | --- | --- | --- | --- | --- | --- |
| 1 | *ADIPOQ* | 1.28e-05 | 1.01e-05,1.57e-05 | 27.450 | 2.100 | chr3:186572031(10bp del) |
| 2 | *DEFA5* | 1.77e-05 | 1.32e-05,2.25e-05 | 21.300 | 2.100 | chr8:6914214 |
| 3 | *CRTAP* | 4.39e-05 | 3.38e-05,5.47e-05 | 18.560 | 2.100 | chr3:33156025 |
| 4 | *SUSD5* | 6.1e-05 | 4.91e-05,7.36e-05 | 21.010 | 2.100 | chr3:33260201 |
| 5 | *SCN10A* | 0.000186 | 0.000144,0.00023 | 16.650 | 2.100 | chr3:38798314 |
| 6 | *TCEA3* | 0.00155 | 0.00121,0.0019 | 13.280 | 0.000 | chr1:23724330 |
| 7 | *USH1G* | 0.00159 | 0.00124,0.00195 | 12.920 | 0.000 | chr17:72915919 |
| 8 | *FURIN* | 0.0016 | 0.00126,0.00197 | 12.490 | 0.000 | chr15:91419548 |
| 9 | *FBXL17* | 0.00162 | 0.00128,0.00199 | 16.030 | 0.000 | chr5:107716997(9bp del) |
| 10 | *ATP7A* | 0.00164 | 0.00129,0.00201 | 11.980 | 0.000 | chrX:77271271 |
| 11 | *NOX1* | 0.00168 | 0.00133,0.00206 | 12.490 | 0.000 | chrX:100117243 |
| 12 | *TFAP2D* | 0.00172 | 0.00136,0.0021 | 12.340 | 0.000 | chr6:50681803 |
| 13 | *ALG1L2* | 0.00172 | 0.00136,0.0021 | 9.740 | 0.000 | chr3:129811999 |
| 14 | *SEC31A* | 0.00174 | 0.00138,0.00212 | 13.870 | 0.000 | chr4:83801961(3bp del) |
| 15 | *B4GALT2* | 0.00174 | 0.00138,0.00212 | 7.100 | 0.000 | chr1:44447579 |
| 16 | *PTP4A3* | 0.00176 | 0.0014,0.00214 | 13.280 | 0.000 | chr8:142441127 |
| 17 | *POU1F1* | 0.00178 | 0.00142,0.00216 | 13.870 | 0.000 | chr3:87309197(3bp del) |
| 18 | *CC2D2B* | 0.00178 | 0.00142,0.00216 | 12.880 | 0.000 | chr10:97786973 |
| 19 | *SSX2IP* | 0.00178 | 0.00142,0.00216 | 11.840 | 0.000 | chr1:85124142 |
| 20 | *UGCG* | 0.00178 | 0.00142,0.00216 | 9.310 | 0.000 | chr9:114676970 |
| 21 | *LOC100505836* | 0.0018 | 0.00143,0.00219 | 17.760 | 0.000 | chr3:22175400(1bp del) |
| 22 | *SIPA1* | 0.0018 | 0.00143,0.00219 | 11.840 | 0.000 | chr11:65417258 |
| 23 | *GPR137* | 0.00188 | 0.0015,0.00227 | 13.280 | 0.000 | chr11:64056551 |
| 24 | *ANKRD13D* | 0.00188 | 0.0015,0.00227 | 11.500 | 0.000 | chr11:67067041 |
| 25 | *SLC22A7* | 0.0019 | 0.00152,0.00229 | 14.770 | 0.000 | chr6:43272444 |

**Supplementary Table 2. Top Ranked Genes from PHEVOR Analysis (HPO terms: HP:0000819 (Diabetes), HP:0005978 (Type 2 diabetes mellitus), HP:0000077 (Abnormality of the kidney), HP:0003774 (Stage 5 chronic kidney disease), and HP:0000112 (Nephropathy)).**

| **RANK** | **GENE** | **PHEVOR_SCORE** | **PHEVOR_PRIOR** | **PHEVOR_RAWSCR** | **ORIG_P** | **PHEVOR _P** |
| --- | --- | --- | --- | --- | --- | --- |
| 1 | *ADIPOQ* | 5.404844643 | 0.764779128 | 0.109751582 | 1.00E-05 | 3.00E-06 |
| 2 | *CRTAP* | 4.767205492 | 0.719774628 | 0.076210967 | 4.00E-05 | 1.00E-05 |
| 3 | *DEFA5* | 4.612232448 | 0.420219951 | 0.027952301 | 1.00E-05 | 2.00E-05 |
| 4 | *FN1* | 4.299374968 | 0.976920484 | 3.485219096 | 0.00212 | 5.00E-05 |
| 5 | *IFT172* | 3.626384351 | 0.991709948 | 7.174059286 | 0.0275 | 0.000236327 |
| 6 | *C2orf71* | 3.452943857 | 0.9284174 | 2.512572752 | 0.00455 | 0.000352292 |
| 7 | *PROM1* | 3.450920063 | 0.945115183 | 2.560136781 | 0.00606 | 0.000353937 |
| 8 | *SCN10A* | 3.443400715 | 0.340547639 | 0.022364205 | 0.000186 | 0.000360116 |
| 9 | *UMOD* | 3.43580807 | 0.99027871 | 5.85741014 | 0.036 | 0.000366465 |
| 10 | *BBS12* | 3.426669849 | 0.958155356 | 2.700027696 | 0.0085 | 0.000374255 |
| 11 | *CFH* | 3.417546154 | 0.913468909 | 1.435535773 | 0.00402 | 0.000382197 |
| 12 | *ATP7A* | 3.393234708 | 0.802468409 | 0.177141056 | 0.00164 | 0.000404194 |
| 13 | *MARS* | 3.354964708 | 0.822346722 | 0.270874363 | 0.00204 | 0.000441411 |
| 14 | *RECQL4* | 3.327181839 | 0.95751925 | 2.674502653 | 0.0105 | 0.000470559 |
| 15 | *LIG4* | 3.31070913 | 0.964993496 | 2.907723131 | 0.0133 | 0.000488741 |
| 16 | *SLC12A3* | 3.309732253 | 0.982645438 | 4.417402159 | 0.027 | 0.000489841 |
| 17 | *TNXB* | 3.306213766 | 0.808382372 | 0.204577775 | 0.00208 | 0.000493823 |
| 18 | *SUSD5* | 3.304674703 | 0.109556331 | 0.007725537 | 6.00E-05 | 0.000495576 |
| 19 | *GJB3* | 3.300793492 | 0.933824301 | 2.527988474 | 0.00701 | 0.000500022 |
| 20 | *ANKS6* | 3.271190201 | 0.901541921 | 1.003191344 | 0.00488 | 0.000535275 |
| 21 | *HLA-DRB1* | 3.267653989 | 0.979782961 | 3.703701641 | 0.0255 | 0.000539649 |
| 22 | *GTF2I* | 3.249178245 | 0.983758623 | 4.516509535 | 0.033 | 0.000563089 |
| 23 | *LRIG2* | 3.246896307 | 0.874189362 | 0.581101262 | 0.00392 | 0.000566054 |
| 24 | *BLK* | 3.245657647 | 0.989165524 | 5.67989304 | 0.0493 | 0.00056767 |
| 25 | *PARN* | 3.208842155 | 0.947341554 | 2.577328684 | 0.011 | 0.000617859 |

**Supplementary Table 3. Top Ranked Genes from PHEVOR Analysis (HPO terms: HP:0000819 (Diabetes) and HP:0005978 (Type 2 diabetes mellitus)).**

| **RANK** | **GENE** | **PHEVOR_SCORE** | **PHEVOR_PRIOR** | **PHEVOR_RAWSCR** | **ORIG_P** | **PHEVOR _P** |
| --- | --- | --- | --- | --- | --- | --- |
| 1 | *ADIPOQ* | 6.31134831 | 0.963256697 | 1.727846879 | 1.00E-05 | 4.00E-07 |
| 2 | *DEFA5* | 5.2371406 | 0.753438076 | 0.487527125 | 1.00E-05 | 5.00E-06 |
| 3 | *CRTAP* | 4.313245966 | 0.474537926 | 0.266507723 | 4.00E-05 | 4.00E-05 |
| 4 | *ATP7A* | 4.221285858 | 0.964717635 | 1.818481053 | 0.00164 | 6.00E-05 |
| 5 | *PROM1* | 4.056291641 | 0.985796893 | 3.47380911 | 0.00606 | 8.00E-05 |
| 6 | *FN1* | 3.990952491 | 0.954143222 | 1.394285851 | 0.00212 | 0.000102095 |
| 7 | *C2orf71* | 3.921502783 | 0.974457226 | 2.803897084 | 0.00455 | 0.000119797 |
| 8 | *HIP1R* | 3.917455747 | 0.946142844 | 1.235044045 | 0.00212 | 0.000120918 |
| 9 | *LIG4* | 3.912872782 | 0.991014531 | 4.490203428 | 0.0133 | 0.000122201 |
| 10 | *PARN* | 3.855164288 | 0.987605675 | 3.664316758 | 0.011 | 0.000139565 |
| 11 | *FURIN* | 3.823184234 | 0.914280467 | 0.919114904 | 0.0016 | 0.000150228 |
| 12 | *UCN* | 3.804523574 | 0.932437847 | 1.073507907 | 0.00216 | 0.000156822 |
| 13 | *OPRM1* | 3.783145534 | 0.925411428 | 1.008783512 | 0.00204 | 0.000164734 |
| 14 | *CHRNA7* | 3.77271172 | 0.960056545 | 1.58257741 | 0.00404 | 0.000168739 |
| 15 | *GJB3* | 3.77199101 | 0.97661385 | 2.906687651 | 0.00701 | 0.000169019 |
| 16 | *SCN10A* | 3.743338483 | 0.50744383 | 0.284458736 | 0.000186 | 0.000180544 |
| 17 | *POU4F2* | 3.6601226 | 0.940577363 | 1.161085414 | 0.00345 | 0.000218667 |
| 18 | *HLA-DRB1* | 3.631619445 | 0.991153668 | 4.554273171 | 0.0255 | 0.000233496 |
| 19 | *SLC12A3* | 3.579385263 | 0.99059712 | 4.398281931 | 0.027 | 0.00026333 |
| 20 | *SUSD5* | 3.575233604 | 0.18659387 | 0.135007984 | 6.00E-05 | 0.000265859 |
| 21 | *ZMPSTE24* | 3.554457005 | 0.960682662 | 1.606982804 | 0.00677 | 0.000278883 |
| 22 | *PTPN11* | 3.55268377 | 0.965552457 | 1.887401497 | 0.00779 | 0.000280024 |
| 23 | *NOX1* | 3.547028996 | 0.855703783 | 0.679374782 | 0.00168 | 0.000283692 |
| 24 | *IFT172* | 3.545975528 | 0.990040572 | 4.172698338 | 0.0275 | 0.000284381 |
| 25 | *FLT4* | 3.53998081 | 0.880470172 | 0.759628046 | 0.00212 | 0.000288333 |

**Supplementary Table 4. Top Ranked Genes from PHEVOR Analysis (HPO terms: HP:0000077 (Abnormality of the kidney), HP:0003774 (Stage 5 chronic kidney disease), and HP:0000112 (Nephropathy)).**

| **RANK** | **GENE** | **PHEVOR_SCORE** | **PHEVOR_PRIOR** | **PHEVOR_RAWSCR** | **ORIG_P** | **PHEVOR _P** |
| --- | --- | --- | --- | --- | --- | --- |
| 1 | *ADIPOQ* | 5.431441929 | 0.775617035 | 0.090460907 | 1.00E-05 | 3.00E-06 |
| 2 | *CRTAP* | 4.840887212 | 0.752688427 | 0.07532898 | 4.00E-05 | 1.00E-05 |
| 3 | *DEFA5* | 4.594707363 | 0.410421417 | 0.022872202 | 1.00E-05 | 2.00E-05 |
| 4 | *FN1* | 4.569308263 | 0.9874698 | 3.480384354 | 0.00212 | 2.00E-05 |
| 5 | *CFH* | 3.677544462 | 0.950518736 | 1.435287209 | 0.00402 | 0.00021007 |
| 6 | *UMOD* | 3.668768888 | 0.994291534 | 5.857905486 | 0.036 | 0.000214357 |
| 7 | *BBS12* | 3.640730833 | 0.974015823 | 2.698506618 | 0.0085 | 0.000228649 |
| 8 | *IFT172* | 3.541271533 | 0.989933204 | 3.917140782 | 0.0275 | 0.000287477 |
| 9 | *RECQL4* | 3.535258129 | 0.973257852 | 2.661931424 | 0.0105 | 0.000291484 |
| 10 | *ANKS6* | 3.479548254 | 0.936685774 | 1.003092891 | 0.00488 | 0.000331366 |
| 11 | *ATP7A* | 3.425795891 | 0.81408404 | 0.160360139 | 0.00164 | 0.000375009 |
| 12 | *MARS* | 3.414087852 | 0.841370979 | 0.268944808 | 0.00204 | 0.000385252 |
| 13 | *AGXT* | 3.412318734 | 0.987848785 | 3.535409983 | 0.0305 | 0.000386824 |
| 14 | *SCN10A* | 3.395895758 | 0.316427426 | 0.017622086 | 0.000186 | 0.000401726 |
| 15 | *LRIG2* | 3.394776066 | 0.907124923 | 0.580930885 | 0.00392 | 0.000402762 |
| 16 | *CFHR5* | 3.364281108 | 0.970036477 | 2.541621182 | 0.0138 | 0.000432047 |
| 17 | *TNXB* | 3.350693203 | 0.823748164 | 0.203666544 | 0.00208 | 0.000445772 |
| 18 | *KANK2* | 3.262898758 | 0.89537638 | 0.516255192 | 0.00465 | 0.000545587 |
| 19 | *PIK3C2A* | 3.224754975 | 0.813326069 | 0.158592437 | 0.00259 | 0.000595643 |
| 20 | *OPRM1* | 3.057087526 | 0.699819982 | 0.053045662 | 0.00204 | 0.000876056 |
| 21 | *HIP1R* | 3.057043451 | 0.707833642 | 0.055584508 | 0.00212 | 0.000876145 |
| 22 | *SUSD5* | 3.053842986 | 0.064595014 | 0.004268778 | 6.00E-05 | 0.00088262 |
| 23 | *CLDN19* | 3.015369007 | 0.988796248 | 3.65855067 | 0.0785 | 0.0009643 |
| 24 | *UCN* | 3.010104302 | 0.689018902 | 0.050354392 | 0.00216 | 0.000976049 |
| 25 | *FURIN* | 2.994299636 | 0.612653371 | 0.03874582 | 0.0016 | 0.001012187 |
