# Supplementary material for: A dominant negative ADIPOQ mutation in a diabetic family with renal disease, hypoadiponectinemia, and hyperceramidemia
Source: NPJ Genom Med. 2022 Jul 22;7:43. doi: 10.1038/s41525-022-00314-z (PMC9307825; doi:10.1038/s41525-022-00314-z)
Supplement: Supplementary file 2 — Reporting Summary [file 41525_2022_314_MOESM2_ESM.pdf]

nature portfolio

Corresponding author(s): Marcus G. Pezzolesi, William L. Holland

Last updated by author(s): Mar 19, 2022

Reporting Summary

Nature Portfolio wishes to improve the reproducibility of the work that we publish. This form provides structure for consistency and transparency in reporting. For further information on Nature Portfolio policies, see our Editorial Policies and the Editorial Policy Checklist.

Statistics

For all statistical analyses, confirm that the following items are present in the figure legend, table legend, main text, or Methods section.

n/a	Confirmed	
☐	☒	The exact sample size (*n*) for each experimental group/condition, given as a discrete number and unit of measurement
☐	☒	A statement on whether measurements were taken from distinct samples or whether the same sample was measured repeatedly
☐	☒	The statistical test(s) used AND whether they are one- or two-sided<br>*Only common tests should be described solely by name; describe more complex techniques in the Methods section.*
☒	☐	A description of all covariates tested
☐	☒	A description of any assumptions or corrections, such as tests of normality and adjustment for multiple comparisons
☐	☒	A full description of the statistical parameters including central tendency (e.g. means) or other basic estimates (e.g. regression coefficient) AND variation (e.g. standard deviation) or associated estimates of uncertainty (e.g. confidence intervals)
☐	☒	For null hypothesis testing, the test statistic (e.g. *F*, *t*, *r*) with confidence intervals, effect sizes, degrees of freedom and *P* value noted<br>*Give P values as exact values whenever suitable.*
☒	☐	For Bayesian analysis, information on the choice of priors and Markov chain Monte Carlo settings
☒	☐	For hierarchical and complex designs, identification of the appropriate level for tests and full reporting of outcomes
☒	☐	Estimates of effect sizes (e.g. Cohen's *d*, Pearson's *r*), indicating how they were calculated

Our web collection on statistics for biologists contains articles on many of the points above.

Software and code

Policy information about availability of computer code

Data collection	N/A
Data analysis	Sention Genomics DNASeq software was used for WGS data analysis, pVAAST and PHEVOR were used for unified linkage analysis and rare variant association testing, all other statistical analyses were performed using SAS.

For manuscripts utilizing custom algorithms or software that are central to the research but not yet described in published literature, software must be made available to editors and reviewers. We strongly encourage code deposition in a community repository (e.g. GitHub). See the Nature Portfolio guidelines for submitting code & software for further information.

Data

Policy information about availability of data

All manuscripts must include a data availability statement. This statement should provide the following information, where applicable:

- Accession codes, unique identifiers, or web links for publicly available datasets
- A description of any restrictions on data availability
- For clinical datasets or third party data, please ensure that the statement adheres to our policy

The data that support the findings of this study are available from the corresponding author upon request.

Field-specific reporting

Please select the one below that is the best fit for your research. If you are not sure, read the appropriate sections before making your selection.

☒ Life sciences ☐ Behavioural & social sciences ☐ Ecological, evolutionary & environmental sciences

For a reference copy of the document with all sections, see nature.com/documents/nr-reporting-summary-flat.pdf

Life sciences study design

All studies must disclose on these points even when the disclosure is negative.

Sample size	Samples size was determined based on available members of the family that was studied.
Data exclusions	No data were excluded.
Replication	N/A
Randomization	N/A
Blinding	N/A

Reporting for specific materials, systems and methods

We require information from authors about some types of materials, experimental systems and methods used in many studies. Here, indicate whether each material, system or method listed is relevant to your study. If you are not sure if a list item applies to your research, read the appropriate section before selecting a response.

Materials & experimental systems

n/a	Involved in the study	
☐	☒	Antibodies
☐	☒	Eukaryotic cell lines
☒	☐	Palaeontology and archaeology
☒	☐	Animals and other organisms
☐	☒	Human research participants
☒	☐	Clinical data
☒	☐	Dual use research of concern

Methods

n/a	Involved in the study	
☒	☐	ChIP-seq
☒	☐	Flow cytometry
☒	☐	MRI-based neuroimaging

Antibodies

Antibodies used	Rabbit polyclonal anti-adiponectin (Abcam, ab75989), goat-anti-rabbit AlexaFluor Plus 680 (Invitrogen), Mouse monoclonal anti-beta-actin (Cell Signaling, 3700S), Alexa-fluor Plus 488 Anti-Mouse (ThermoFisher, A32723) and Alexa-fluor Plus 800 Anti-Rabbit (ThermoFisher, A32735)
Validation	*Describe the validation of each primary antibody for the species and application, noting any validation statements on the manufacturer's website, relevant citations, antibody profiles in online databases, or data provided in the manuscript.*

Eukaryotic cell lines

Policy information about cell lines

Cell line source(s)	HEK293T
Authentication	These cell have been used extensively by the Holland lab and validated previously.
Mycoplasma contamination	All cells tested negative for mycoplasma.
Commonly misidentified lines (See ICLAC register)	*Name any commonly misidentified cell lines used in the study and provide a rationale for their use.*

Human research participants

Policy information about studies involving human research participants

Population characteristics	The present study investigated members of a family of European ancestry enriched for diabetes and end-stage renal disease (ESRD) from the Joslin Study of Genetics of Nephropathy in Type 2 Diabetes Family Collection.
Recruitment	The recruitment of the Joslin Study of Genetics of Nephropathy in Type 2 Diabetes Family Collection has previously been described. Briefly, between 1993 and 2003, families with an apparent autosomal dominant mode of inheritance of type 2 diabetes (T2D), irrespective of their nephropathy status, were recruited to the Joslin Study of Genetics of Nephropathy in Type 2 Diabetes Family Collection through T2D probands receiving medical care at the Joslin Clinic. All participants provided informed written consent to participate in this study.
Ethics oversight	Committee on Human Subjects of the Joslin Diabetes Center

Note that full information on the approval of the study protocol must also be provided in the manuscript.
